# A Reference-Sampling Based Calibration-Free Fractional-N PLL with a PI-Linked Sampling Clock Generator

**DOI:** 10.3390/s21206824

**Published:** 2021-10-14

**Authors:** Jae-Soub Han, Tae-Hyeok Eom, Seong-Wook Choi, Kiho Seong, Dong-Hyun Yoon, Tony Tae-Hyong Kim, Kwang-Hyun Baek, Yong Shim

**Affiliations:** 1 School of Electrical and Electronics Engineering, Chung-Ang University, Seoul 06974, Korea; lynn2776@cau.ac.kr (J.-S.H.); choiseonguk@cau.ac.kr (S.-W.C.); tjdrlgh@cau.ac.kr (K.S.); kbaek@cau.ac.kr (K.-H.B.); 2 Samsung Electronics, Hwaseong 18448, Korea; th018.eom@samsung.com; 3 School of Electrical and Electronics Engineering, Nanyang Technological University, Singapore 639798, Singapore; donghyun.yoon@ntu.edu.sg (D.-H.Y.); THKIM@ntu.edu.sg (T.T.-H.K.)

**Keywords:** calibration-free, fractional-N, frequency synthesizer, low-phase noise, low power, reference-sampling PLL, sampling clock generator, sub-sampling PLL, phase locked loop

## Abstract

Sampling-based PLLs have become a new research trend due to the possibility of removing the frequency divider (FDIV) from the feedback path, where the FDIV increases the contribution of in-band noise by the factor of dividing ratio square (N^2^). Between two possible sampling methods, sub-sampling and reference-sampling, the latter provides a relatively wide locking range, as the slower input reference signal is sampled with the faster VCO output signal. However, removal of FDIV makes the PLL not feasible to implement fractional-N operation based on varying divider ratios through random sequence generators, such as a Delta-Sigma-Modulator (DSM). To address the above design challenges, we propose a reference-sampling-based calibration-free fractional-N PLL (RSFPLL) with a phase-interpolator-linked sampling clock generator (PSCG). The proposed RSFPLL achieves fractional-N operations through phase-interpolator (PI)-based multi-phase generation instead of a typical frequency divider or digital-to-time converter (DTC). In addition, to alleviate the power burden arising from VCO-rated sampling, a flexible mask window generation method has been used that only passes a few sampling clocks near the point of interest. The prototype PLL system is designed with a 65 nm CMOS process with a chip size of 0.42 mm^2^. It achieves 322 fs rms jitter, −240.7 dB figure-of-merit (FoM), and −44.06 dBc fractional spurs with 8.17 mW power consumption.

## 1. Introduction

Recently, communication-based industries such as home IoT, 5G communications, autonomous vehicles, and mobile high-speed interfaces are growing rapidly [1,2,3,4]. Phase locked loop (PLL)-based clock generators are of particular interest in such applications, where the key characteristics are fine frequency resolution, excellent noise performance, low power consumption, and small chip area.

**Figure 1:**
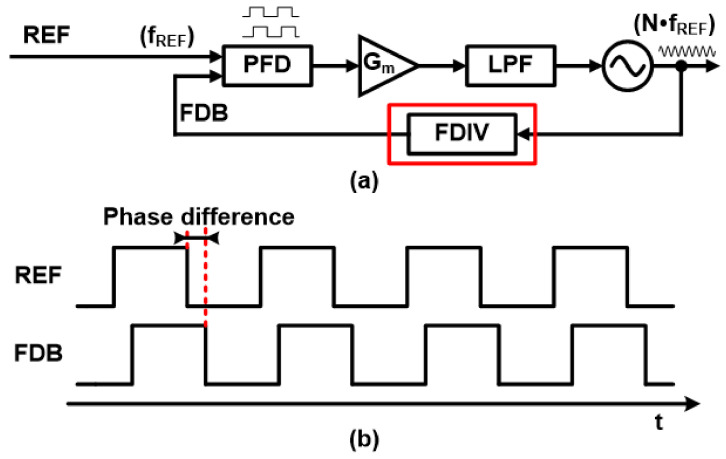
(**a**) Block diagram of conventional charge pump PLL and (**b**) waveform of the reference signal and feedback signal.

The most common frequency synthesizer for these applications is the PLL. A basic block diagram of the classical charging pump PLL (CPLL) is shown in Figure 1a, where the frequency of the VCO output signal varies with the change in the dividing ratio (N) along the feedback path. Since the feedback signal (FDB) of the frequency divider (FDIV) must have the same frequency as the input reference signal (REF), the frequency of the VCO output signal is N∙f_REF_, where f_REF_ represents the frequency of the input reference signal.

If N is an integer, the output frequency is an integer multiple of the input signal, called an integer-N PLL. Even though it has versatile usage, the output signal is only changed by an integer multiple of the REF signal, which is sometimes not acceptable for certain applications that require high-frequency resolution. To address this limited resolution problem, fractional-N PLL has been introduced where the output signal changes with a fractional portion of the f_REF_. The block diagram of a typical fractional-N PLL is approximately the same as an integer-N PLL. An integer type has a fixed dividing ratio N, whereas a fractional-N type has varying divide ratios (N, N + 1) through a control signal. Figure 1b shows the general waveform of the REF and FDB signals. Both signals have rail-to-rail swings and are used to determine phase and frequency differences between the two signals via Phase-Frequency Detector (PFD).

Even with an enhanced frequency resolution from the fractional-N PLL structure, one of the disadvantages of classical PLLs is that the in-band noise of this type of PLL is increasing by the factor of dividing ratio square (N^2^) [5,6,7,8]. These disadvantages push the research community to consider a different phase-frequency comparison method based on sampling, i.e., without using FDIV units. Sampling-based PLLs can be categorized into two groups, sub-sampling PLLs (SSPLLs) [9,10,11] and reference-sampling PLLs (RSPLLs) [12,13,14], depending on the signal used as the sampling clock and the signal to be sampled. For SSPLL, the relatively low-frequency input reference signal sub-samples the fast VCO output signal. On the other hand, the fast VCO output signal samples the slow input reference signal in RSPLL. Even though our proposal refers to the basic structure of RSPLL, it would be beneficial to introduce the various SSPLL and RSPLL types, and the circuit techniques used for each PLL in the succeeding chapter, to help in understanding the details used in our design.

## 2. Sub-Sampling PLL and Reference-Sampling PLL

**Figure 2:**
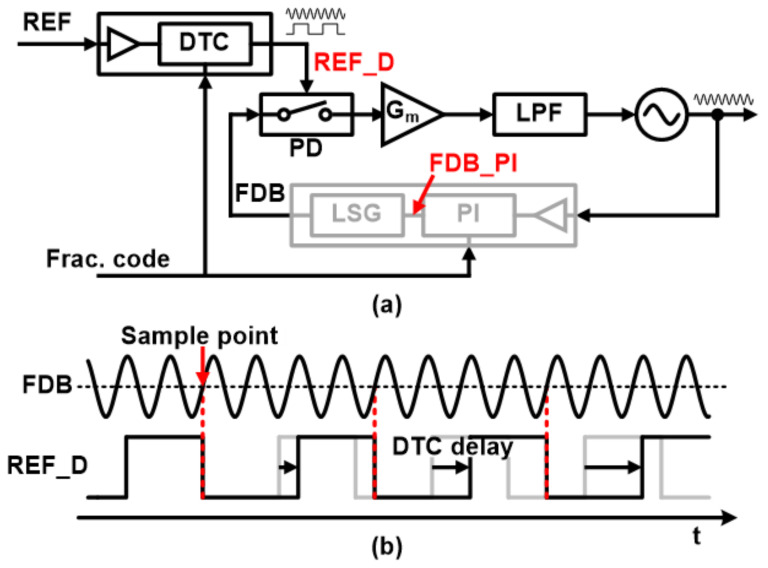
(**a**) Block diagram of sub-sampling fractional-N PLL and (**b**) waveform of the reference signal and feedback signal.

The basic block diagram of SSPLL is shown in Figure 2a. The sub-sampling PLL uses a phase detector (PD) that sub-samples the high-frequency VCO output with a relatively slow REF signal. The PLL structure can improve the in-band noise characteristics by removing the divider. On top of these noise improvements, fractional-N operations based on digital-time converters (DTC) have been proposed to achieve fine frequency resolution [15,16,17,18,19].

SSPLL uses a DTC to insert different delays into the REF signal to mimic the frequency difference between the REF signal and the FDB signal. Figure 2b illustrates the basic concept of fractional-N operations in SSPLL. The DTC provides various delays for each rising edge of the REF signal. Therefore, the two signals become fractional multiple frequency relationships. However, there are two issues with the DTC-based fractional-N implementation that arise from the genuine properties of the DTC unit. First, DTC gain is very sensitive to the variations in PVTs and also has nonlinearity problems [17]. Hence, an additional calibration logic is typically required which makes the design expensive. The second issue comes from the required resolution of the DTC in SSPLL. Here, the DTC unit is supposed to cover a short delay range to maintain the lock within the linear region of the VCO output signal.

**Figure 3:**
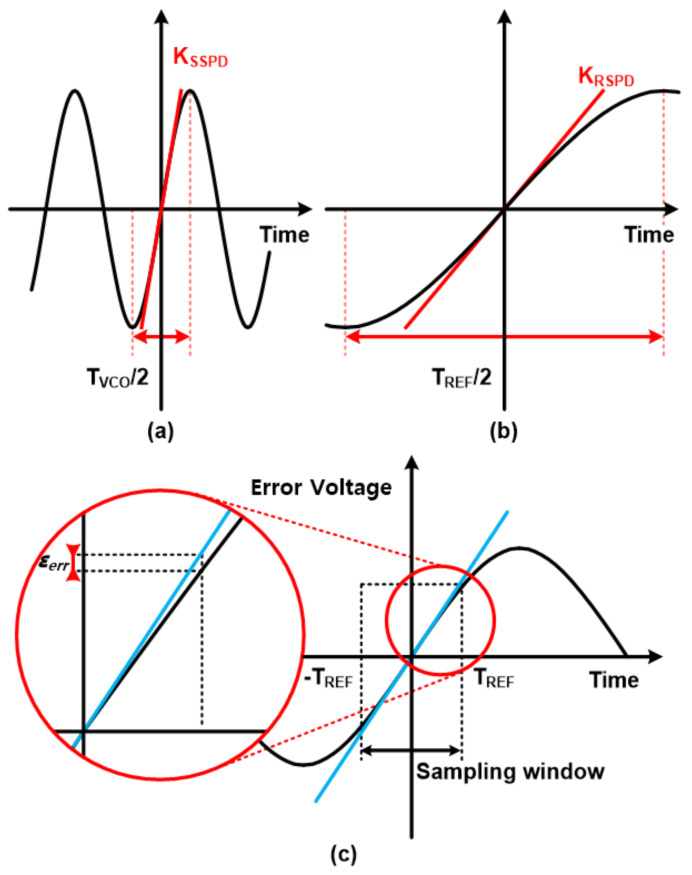
(**a**) Locking range of sub-sampling-based PLL and (**b**) reference-sampling-based PLL; (**c**) maximum sampling error of the proposed RSFPLL.

The linear region for locking is shown in Figure 3a. The fast-frequency sine wave represents the VCO output signal to be sampled. The sampling pulse must then sample the sine wave within T_VCO_/2 in the figure. Since the VCO output range is typically a multi-GHz, one cycle period of the VCO signal is very short, and the DTC needs to vary the delay with a fine step within this region for fractional-N operation. This implies that sometimes high-resolution (<1 ps) DTCs are required which is quite challenging. Furthermore, a DTC with a finer step naturally occupies a large silicon area when considering the total delay range need to cover. To alleviate the constraints of DTCs on SSPLL systems, a phase-interpolator (PI)-assisted SSPLL system is proposed in [18].

Here, PI adds various delays to the VCO output signal, so that the frequency of the FDB_PI signal changes over time. Furthermore, after PI units, [20] uses Linear Slope Generator (LSG) to linearize the sampling region. The LSG tilts the slope of the incoming signal from the PI to provide a wider linear region to sample with the REF_D signal.

**Figure 4:**
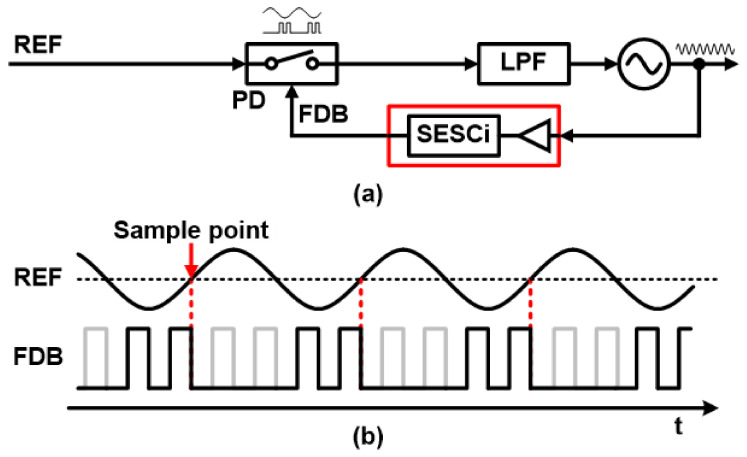
(**a**) Block diagram of conventional reference-sampling PLL and (**b**) waveform of the reference signal and sample edge selection circuit signal.

To alleviate the limitations of the sub-sampling structures, a reference-sampling PLL (RSPLL) is proposed [12]. As the PLL name indicates, the RSPLL uses the VCO output signal as a sampling clock, and the buffered VCO output signal samples the input reference signal to determine the lock condition. A typical block diagram of the RSPLL is shown in Figure 4 with two input waveforms for the PD unit.

This structure offers multiple advantages. First, similar to SSPLL, RSPLLs do not require a frequency divider, which can improve in-band noise characteristics. In addition, the reference sine wave can be used without going through the buffering phase, reducing the noise contribution of the input buffer. The potential power overhead that arises from VCO rate clock sampling can be solved by simple digital logic that passes only a few VCO output pulses for sampling. The sample edge selection circuit (SESCi) unit in Figure 4a selects sampling pulses (VCO output clock) near the zero-crossing point of the input sine wave using a mask pulse. Therefore, the waveform of the sampling clock in Figure 4b is enabled only for a few portions of the one cycle period of the input sine wave.

Another benefit of RSPLL comes from a wide locking range. Compared to SSPLL, RSPLL samples input sine wave signals that are typically much wider (N times) than VCO output signals. The input signal also has a very linear slope near the zero intersection, providing a sufficient locking range. Related waveforms can be seen in Figure 3b.

Finally, it would be beneficial to mention sampling error reduction compared to subsampling methods. Here, the sampling error (*ε_err_*) can be defined as the voltage difference between the value of a sine wave and the ideal linear line at a certain time *T_VCO_* as shown in Figure 3c. Then, the phase error, $\phi_{err}$ can be calculated as

(1) $$\phi_{err,SSPD}=\frac{2\pi }{T_{VCO}}\cdot \varepsilon_{err}=\frac{N\cdot 2\pi }{T_{REF}}\cdot \varepsilon_{err}$$

(2) $$\phi_{err,RSPD}=\frac{2\pi }{T_{REF}}\cdot \varepsilon_{err}$$

where 𝜙*_err,SSPD_* and 𝜙*_err,RSPD_* represents the phase error of sub-sampling PD (SSPD) and reference-sampling PD (RSPD), respectively [15]. T_VCO_ and *T_REF_* are periods of VCO and reference signals, respectively. Once we compare Equations (1) and (2), the phase error of the RSPD is much more insensitive to *ε_err_* from the fact that there is no coefficient N in the phase error ($\phi_{err}$).

Although the basic concept and low-noise characteristics of the reference-sampling structure were introduced in [12], fractional-N operations are not yet included, most likely due to design complexity for combinations of digital sampling logic and fractional-N features. Instead, a fractional-N operation of the RSPLL was recently proposed in [21]. In [21], the fractional-N function is achieved using the traditional clock counter of the feedback path instead of the SESCi logic. The counter here basically generates sampling signals for fractional-N operations at specific intervals based on fractional code reception. However, the counter generates periodic spurs and quantization errors. Instead of using Delta-Sigma-Modulator (DSM) to address this issue, the RSPLL in [21] exploits capacitor-based digital-to-analog converter (CDAC) and necessity calibration logic to counteract periodic spurs generation. This additional DAC and its logic units occupy a large silicon area, not to mention design complexity.

Following the above discussion, here we propose a fractional-N RSPLL (RSFPLL) that has the following properties: (1) Adopt PI-based multi-phase generation to perform efficient fractional-N operations, but do not use DTCs that require significant design effort. Removing the DTC simplifies the design, and the design shows robust operation against possible environmental changes; (2) Adaptive mask window method was proposed to selectively pass only the VCO output pulses of interest as the sampling clocks. 

The rest of this article is organized as follows. Section 3 introduces the proposed reference-sampling PLLs. Section 4 describes the noise analysis of the proposed system, and Section 5 discusses the measurement results. Finally, Section 6 shows the conclusions.

## 3. Architecture

This chapter first introduces the overall structure and basic behavior of the proposed RSFPLL. This is followed by a description of the subblocks.

### 3.1. Architecture Overview

**Figure 5:**
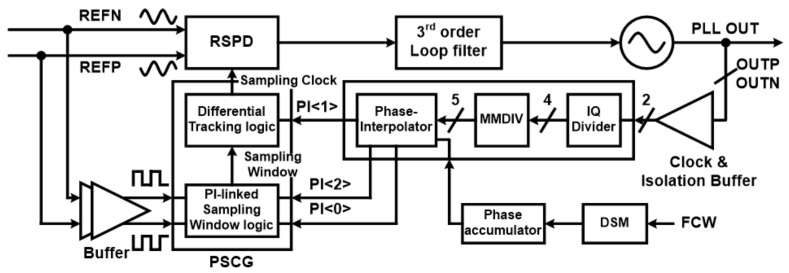
Block diagram of proposed reference-sampling-based calibration-free fractional-N PLL with an PI-linked sampling clock generator.

A block diagram of the proposed reference-sampling fractional-N PLL (RSFPLL) is shown in Figure 5. In the forward path, reference-sampling PD (RSPD), third-order loop filter (LPF), and VCO are connected in series, similar to the conventional RSPLL in [12]. However, along the feedback path of the proposed DTC free fractional-N RSPLL, there are phase-interpolator/multi-modulus divider (PI/MMDIV) and PI-linked sampling clock generator (PSCG).

When the differential VCO output (OUTP, OUTN) is presented to the MMDIV unit after the buffer stage, the IQ divider inside the MMDIV unit generates a four-phase signal. Then, a dual modular multi-phase divider adds an additional phase to generate a five-rotating phase [22]. This 5-phase signal is interpolated into 32 phases through a consecutive 3-bit pipelined PI. Among 32 phase signals, only 3 adjacent signals are selected through DSM code [20] and sent to the following PSCG unit. The PSCG device then creates a sampling window that adaptively changes the activation time so that only the required VCO output pulses pass selectively as the sampling pulses. Sampling pulses generated by the PSCG are used to sample input sine wave inside the reference-sampling phase detector (RSPD), where the sampled output, namely the DC level, is used to adjust the control voltage of the VCO through the LPF unit.

### 3.2. Pipelined Phase-Interpolator with Constant Charge Technique

In the proposed sampling-based PLL structure, PI can provide output signals with multiple phases out of VCO signal, one of which can be arbitrarily selected to perform fractional-N operation without a DTC unit. The PI in our system receives a 5-phase rotation signal from MMDIV and generates 32 interpolated phases using a 3-stage PI. 

**Figure 6:**
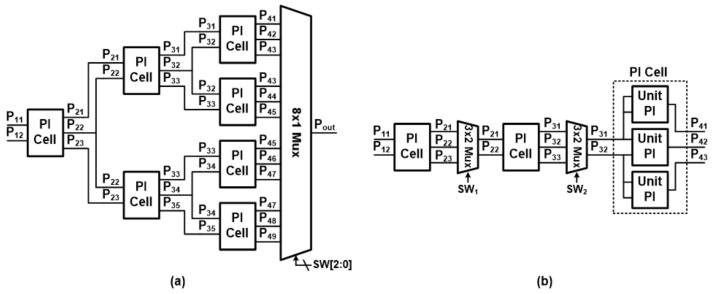
Block diagram of 3-stage (**a**) tournament-type PI and (**b**) pipelined PI with unit PI cell.

In conventional sampling-based fractional PLLs, tournament-type PIs are typically used, and the PI Cells required for N-stage structures are 2^N−1^. Since it might consume large power and area, a pipelined PI using only N + 1 PI Cells for the same PI stages has been proposed [23]. Figure 6a,b show a block diagram for two PI types, each of which consists of three stages. Here the PI Cell receives two input signals with adjacent phases and generates three output signals. The first and last output signals from the PI Cell have the same phase with the two input signals, and the signal at the middle has a phase in between two input signals. For this operation, the PI Cell consists of three separate unit PIs (inset of Figure 6b), each of which receives two inputs and generates an output signal on the center. 

When comparing two PI structures, two types of PI receive two adjacent phase signals (P_11_, P_12_) and generate eight phase signals from the output. Compared to the tournament-type PI that generates all the 8-phase signals, the pipelined PI generates only signals of interest by appropriately selecting two adjacent signals from the previous stage through a 3 × 2 multiplexer. This is how pipelined PI has a small number of PI Cells inside, and we adopted this type of PI for the proposed design.

**Figure 7:**
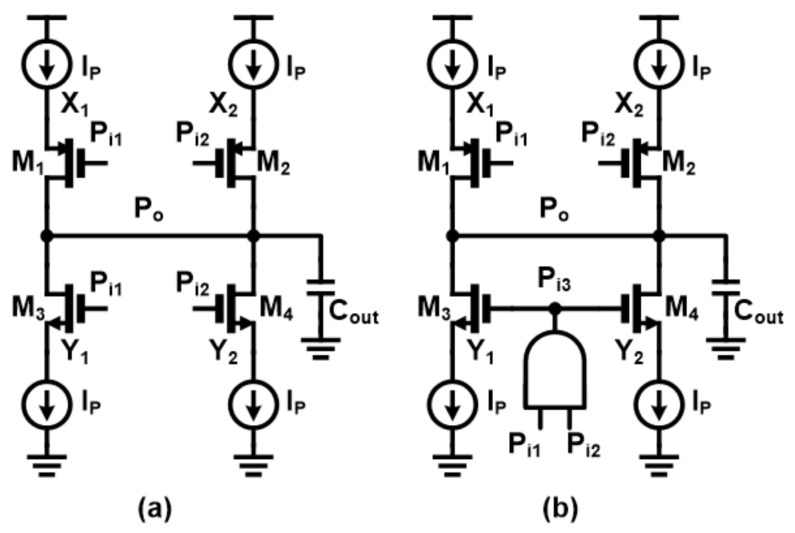
(**a**) Conventional phase-interpolator and (**b**) short-current-free phase-interpolator.

As mentioned, the unit PI receives two input signals and generates an output signal of the phase centered between the two inputs. The conventional unit PI schematic is shown in Figure 7a, which is just another illustration of two inverters with current sources at the top and bottom, and the outputs are tied together to mix the signals.

**Figure 8:**
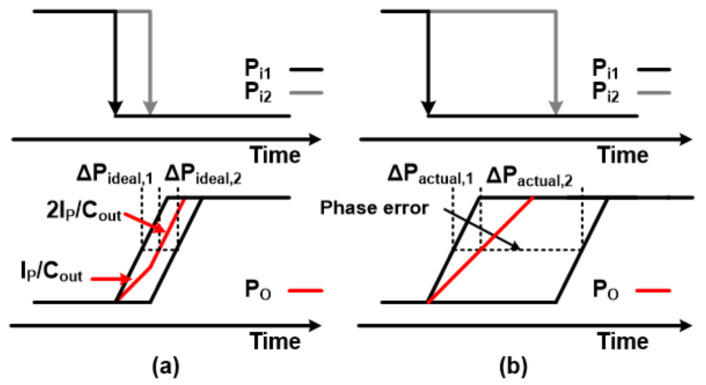
Waveform of (**a**) ideal phase-interpolator and (**b**) when the delays of the two signals show a large difference.

The operation of the conventional unit PI with two inputs P_i1_ and P_i2_ is as follows: (Assume P_i1_ is a fast phase signal) (1) When P_i1_ changes from high to low, the corresponding M1 turns on and M_3_ and M_4_ turn off. (2) Current I_P_ flows through the M_1_ transistor to C_out_ and the output is charged with the slew rate of I_P_/C_out_. (3) Meanwhile, when P_i2_ is switched from high to low, the M_2_ transistor becomes transparent, and the slew rate of the output node doubles (2I_P_/C_out_). These processes are illustrated in Figure 8a. Similarly, reverse operation occurs when P_i1_ and P_i2_ change from low to high, eventually discharging the output capacitor C_out_ to the ground. The conventional PI unit can generate intermediate phases in most cases if the phase difference between the two inputs is not too large, but turning on only one of the NMOS transistors (M_3_ or M_4_) in the middle of operation causes short circuit problems. To avoid this, a circuit technique with a logic AND gate to control the NMOS transistor is proposed in [23], which is shown in Figure 7b. However, if two PI inputs are not close enough, the output signal can be generated with an inappropriate phase. As shown in Figure 8b, if the second PI input signal is too far from the previous signal, the output signal reaches the maximum voltage level before the second input changes. Though this issue can be resolved by reducing the slew rate (I_P_/C_out_), increasing capacitance is not a good idea because capacitors occupy large areas and may need to increase current to maintain a slew rate under normal circumstances.

**Figure 9:**
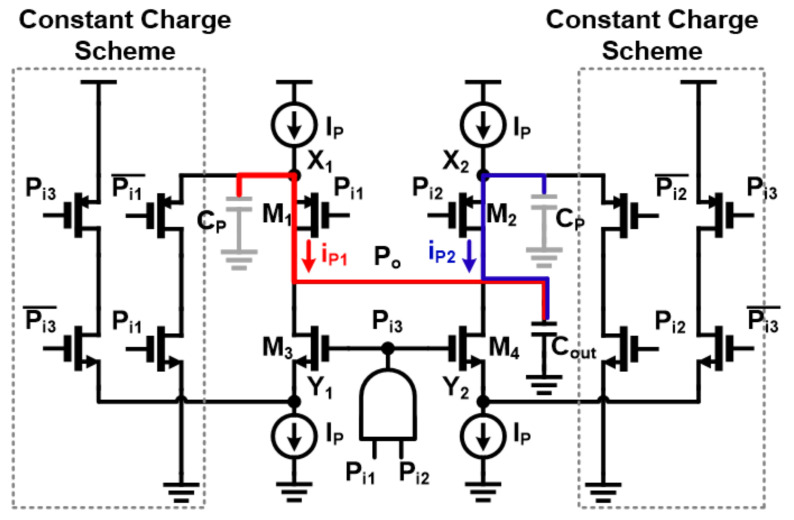
Schematic of unit phase-interpolator with proposed constant charge scheme.

**Figure 10:**
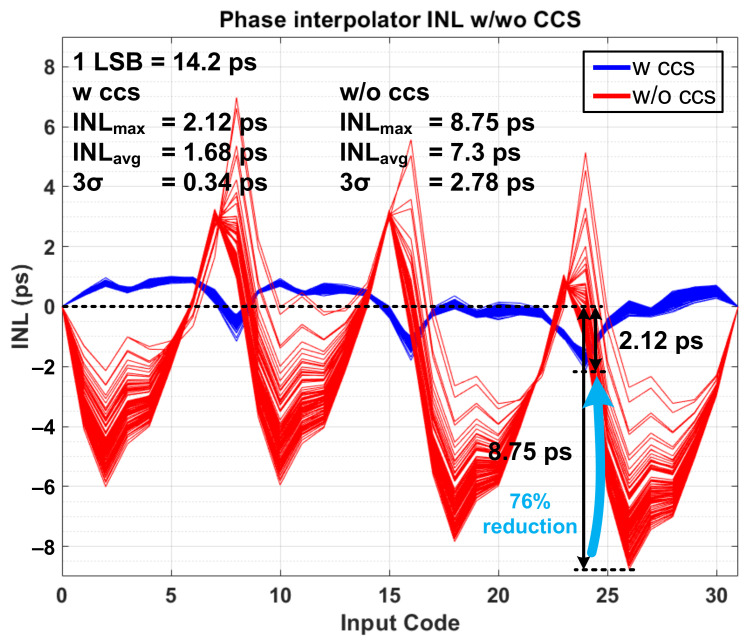
Monte Carlo simulation result of INL characteristics of 5-bit phase-interpolator with constant charge scheme and without constant charge scheme.

To improve the performance of unit PI, we propose constant charge scheme on internal nodes (X_i_, Y_i_). To explain how it works and what has changed from the conventional PI, it would be better to illustrate how a problematic situation occurs when two PI inputs are not close enough. The problem happens by following the next steps: (1) Initially, when both inputs (P_i1_ and P_i2_) are high, M_1_ and M_2_ transistors remain turned off, so charges accumulate on parasitic capacitors of nodes X_1_ and X_2_. Therefore, the voltages of X_1_ and X_2_ rise close to the supply voltage levels. (2) When P_i1_ is changed to low, M_1_ is turned on and the charge of X_1_ immediately moves to the load capacitor at the output which incurs a large current flow. Therefore, the voltage of the output node changes suddenly at the beginning of the PI operation, and the situation gets worse if the second PI input does not arrive within the appropriate time frame. This causes a phase error in the PI output. Additionally, the same problem can occur with M_3_ and M_4_ transistors. To avoid this issue, it is necessary to prevent charging or discharging the source node of each transistor. This means that the source voltage of each transistor should be prepared so that the output voltage does not show a sudden increase/decrease in voltage when the first PI input is received. To this end, constant charge circuits were added to both sides of the unit PI in Figure 7b, making Figure 9. The constant charge circuit then generates a current path to prevent node voltage from charging or discharging. The proposed circuit technique prevents large instantaneous current flows, which leads to improved phase error during normal PI operation. Figure 10 shows the results of post-layout Monte Carlo simulations of 5-bit pipelined PI, especially INL values for all possible input combinations with/without constant charge circuits. The INL values improved by 76% in the worst case as indicated, where the INL values without the constant charge scheme turned out to be 8.75 ps in the worst case and 7.3 ps on average and 2.78 ps in the 3-sigma value, while the INL values in the proposed circuit technique were 2.12 ps in the worst case, 1.68 ps on average and 3-sigma value was 0.34 ps. For reference, the 1 LSB value of 5-bit PI at 2.2 GHz frequency is 14.2 ps. In general, the quantization error of sensitive circuits should be less than 0.5 LSB, which is about 7 ps in the current situation.

### 3.3. PI-Linked Sampling Clock Generator (PSCG)

The RSPLL basically samples input sine waves using VCO-rated high-speed clock signals, which can result in high power consumption. To avoid this problem, sampling of the RSPD is performed only if the voltage level of the input sine wave passes through the DC reference level of the input signal. For this operation, a masking window has been used to selectively choose the sampling pulses using logic AND operation [23]. However, fractional-N operations on the RSPLL architecture require an adaptive masking window whose masking position depends on the PI output signal selected from 32 different phase signals. Here, we propose a concise method to implement a PI-linked sampling clock generation through simple digital logic.

**Figure 11:**
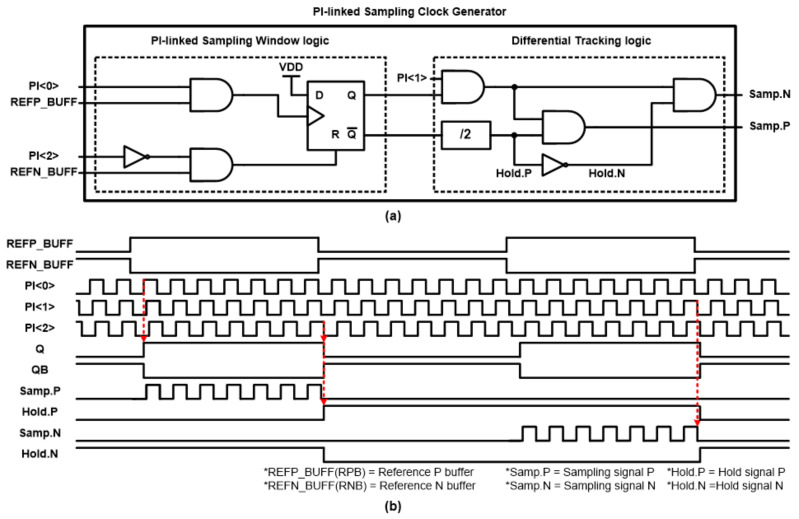
(**a**) Schematic of PI-linked sampling clock generator and (**b**) operation of PI-linked sampling clock generator.

The schematic diagram of the proposed PSCG unit is shown in Figure 11a. The PSCG unit consists of two building blocks, the PI-linked sampling window logic that creates a sampling window for fractional operations and the differential tracking logic. For simple designs, only a few logic gates and resettable DFFs (D flip-flops) were used for both units. The operation of each unit will be followed by where the expected waveforms are depicted in Figure 11b.

When three output signals from the PI arrive, the first phase signal (PI <0>) and the last phase signal (PI <2>) are used as clock and reset signals for the DFF, respectively. Data input ‘D’ receives a fixed VDD input. Therefore, when the PI <0> becomes ‘H’ and RPB is ‘H’ at the same time, the output of the DFF becomes ‘H’. The DFF output remains ‘H’ until the combined signal ‘/PI <2> AND RNB’ becomes ‘H’. In this way, the masking signal, so ‘flexible window’ encapsulates the intermediate input signal PI <1>, and the sampling clock, will be generated by ANDing two receiving signals from the differential tracking logic. This is how the proposed RSPLL can leverage PI and adaptive mask window generation to perform energy efficient fractional-N operations.

**Figure 12:**
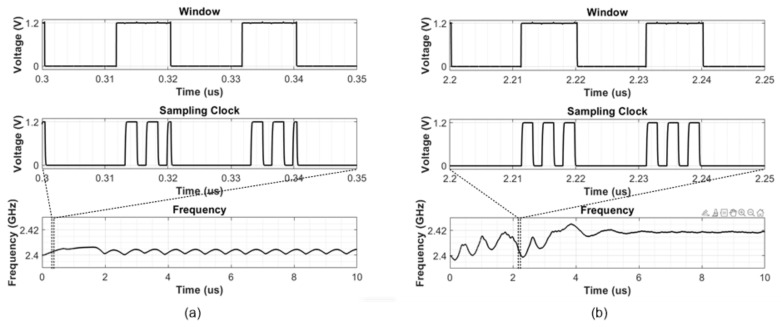
Simulation result of (**a**) conventional sampling clock generator and (**b**) proposed sampling clock generator.

To show the effectiveness of the proposed PSCG method, we compared two cases of fractional-N RSPLL with the first case using a fixed mask window and the second case having a moving window. The simulation waveforms of the two different examples are shown in Figure 12. Figure 12a illustrates the simulation results of the conventional sampling clock generator using the fixed window method proposed in [12], and Figure 12b illustrates the results of the proposed sampling clock generator. As expected, the sample clock generated from the fixed window case has a truncated waveform (Figure 12a) which can occur if three-phase signals out of PI are located at the end of the fixed mask window. As a result, the system samples the wrong points of the input sine wave (far from the input reference DC level), resulting in locking errors.

On the other hand, the proposed sample pulse generation of RSFPLL shows complete consecutive pulses based on the moving mask pulse (Figure 12b). Therefore, the system displays frequency locking after ~6 us. Note that the proposed RSFPLL includes a differential RSPD to reduce charge injection from the sampling switch. Three sampling pulses are required to drive the differential type RSPD, where the first and last pulses are used to generate the sampling window and the intermediate pulse is used as the sampling clock.

### 3.4. Reference-Sampling Phase Detector and VCO

**Figure 13:**
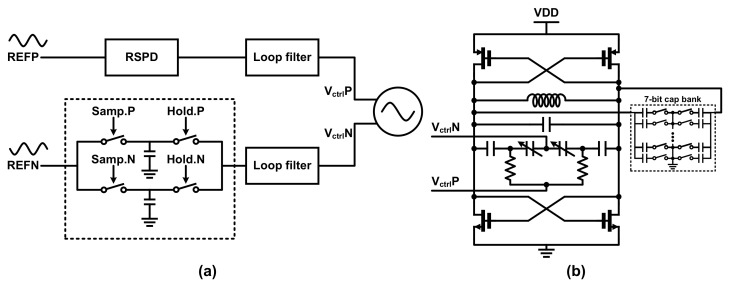
(**a**) RSPD with half-sampling rate circuit and (**b**) differential cross-coupled LC VCO with 7-bit cap bank.

The RSPD of the proposed RSFPLL adopts a differential structure to solve the charge injection problem of the sampling switch. There are two half-sampling rate circuits inside the RSPD to reduce the reference spurs [12]. Figure 13a shows the detailed diagram of the half-sampling rate circuit inside the RSPD unit along with the main components in the forward signal path of the proposed RSFPLL. Here, the RSPD unit receives four control signals (Samp.N, Samp.P, Hold.N, and Hold.P) from the PSCG unit shown in Figure 11. Since the operations of the upper RSPD and the lower RSPD are the same, the operations of the lower RSPD will be briefly described based on the control signal waveform of Figure 11b. Initially, the state of Hold.P = ‘H’ and Hold.N = ‘L’, which makes the signal captured by the lower capacitor, is presented to the next stage loop filter. Meanwhile, the input REFN signal is sampled from the top capacitor based on the sampling input signal (Samp.P). After this sampling period, the REFN signal is captured through the Samp.N signal at the bottom capacitor, and the values stored in the upper capacitor are transferred to the loop filter based on inverse Hold.P and Hold.N inputs. In this way, the RSPD of our system can provide a seamless control signal to the VCO while operates at half-rate speed. Apart from the basic structure, the transmission gate switch was used in the design to reduce fractional spurs. In sampling-based PLLs, linearity must be guaranteed near the DC offset voltage of the input signal. Case studies based on both single transistor and transmission gate switches show that the latter case shows sufficient linearity to perform fractional-N sampling operation.

VCO is implemented in a differential cross-coupled LC structure to receive a differential control voltage. Figure 13b shows the schematic diagram of differential cross-coupled LC VCO in the proposed system. Note that an inductor inside was designed to have an inductance of 2.6 nH, and it was found that the simulated Q-factor is 15.4 at 2.4 GHz. In the reference-sampling structure, the K_VCO_ value is designed at 25 MHz/V to reliably lock to the desired frequency without a frequency lock loop (FLL). Since the output frequency range of the VCO is limited by the low K_VCO_ value, signals of other frequencies that may cause harmonic locking are not generated. A 7-bit binary capacitor bank was added to ensure that the proposed RSFPLL achieves a sufficient frequency synthesis range while maintaining this characteristic.

## 4. Noise Analysis

**Figure 14:**
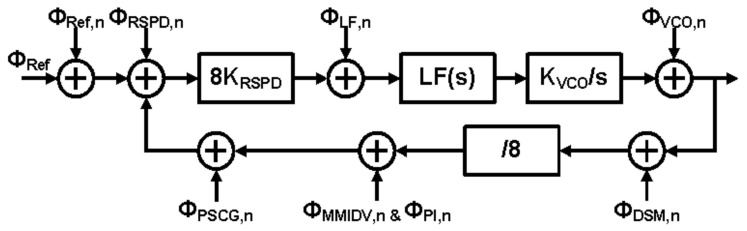
Linear phase domain model of proposed RSFPLL.

This section describes the noise analysis of the proposed RSFPLL using the linear phase domain model in Figure 14. The model includes influences from key sources of noise from PLL components such as RSPD, loop filter (LF), VCO, Delta-Sigma-Modulator (DSM), and PI. It is worth noting that noise components of DSM, PI, and reference inputs have less impact on total phase noise output than those of RSPD and VCO.

For RSPD phase noise, a method from the references [10,12] could be used where the thermal noise current applied to the RSPD sampling capacitor (*C_samp_*) appears as a kT/C noise. Noise sampled by RSPD during the tracking and holding period is stored in *C_samp_*. The RSPD updates the control voltage across the *C_samp_* every reference cycle, so switching noise is also captured and stored in the *C_samp_*. Therefore, it is very important to analyze the phase noise of RSPD accurately. The RSPD phase noise equation from [12] is as follows:

(3) $$L_{in-band,RSPD}\left(f\right)=\frac{1}{2}\cdot \frac{S_{v_{n},RSPD}\left(f\right)}{K_{RSPD}^{2}}=\frac{2kT}{C_{samp}\cdot f_{REF}}\cdot {\left(\frac{N}{2A_{REF}}\right)}^{2}$$

where $S_{v_{n},RSPD}\left(f\right)$ is kT/C noise of RSPD. Based on Equation (3), the RSPD in-band phase noise performance is calculated to be −134 dBc/Hz using a 6 pF sampling capacitor in the proposed design.

Regarding the noise components of the feedback path, the proposed RSFPLL contains MMDIV/PI pairs and PSGC in the feedback path instead of a counter-based divider. The MMDIV and PSCG units consist of logical units (e.g., combinational logic and flip-flops), and PI basically operates as a chain of inverters. Therefore, the main signal passes through mostly logical units, and the noise contribution of these logical units can be ignored [24,25]. In addition, flip-flop (DFF of our design) rearranges the received data based on the reference clock, which in turn removes jitter accumulated in the input data across the various combination logic gates. In particular, according to a study by [24], the noise components by logic gates and DFF are less than −140 dBc, which is sufficiently small compared to the typical RSPD noise in Equation (3). Therefore, jitter accumulation from the conventional divider can be eliminated, and it can be thought that the noise element caused by the divider occurs only in a single DFF. For other noises, PI resolution in our design is equal to 1/32 times 2T_VCO_. Based on this, quantitative analysis shows that the proposed design has 24 dB lower quantization noise compared to the conventional fractional-N divider [20]. Overall, the sum of in-band noise sources except for VCO (which will be explained in the succeeding paragraph) has been found to be −134 dBc/Hz, which is sufficient performance for most targeted applications.

The RSFPLL proposed in this paper is a type-I PLL that does not have a charging pump (CP) in the loop. That is, RSFPLL in this article suppresses noise at −20 dB/decade within the bandwidth, while conventional SSPLL suppresses noise at −40 dB/decade. Therefore, noise generated by VCO has the greatest impact on the overall noise performance of the PLL, and LC-VCO becomes crucial. In the proposed structure, the inductor has a Q factor of 15.4 and an inductance of 2.59 nH. The noise performance of the VCO is shown to be −126.2 dBc/Hz at 1 MHz offset. Finally, we apply a third-order loop filter to the proposed system to shape the out-band noise of MASH 1-1-1 DSM [16].

## 5. Measurement

**Figure 15:**
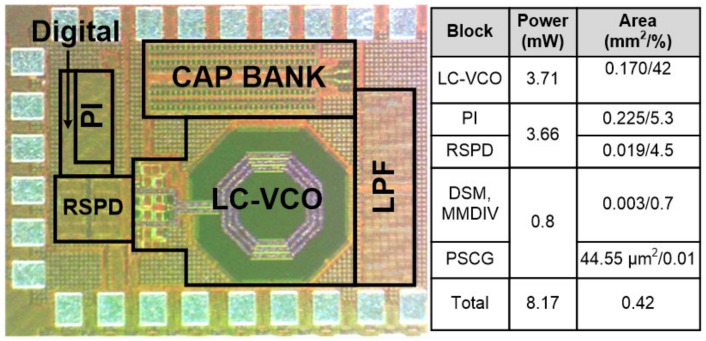
Die photo of proposed RSFPLL and power break down.

The proposed RSFPLL prototype was fabricated with a 65 nm CMOS process with a core area of 0.42 mm^2^. Figure 15 shows a die photo of the proposed RSFPLL (left) and a power and area analysis table (right). Note that the core idea of fractional-N operation lies in the PSCG unit, which accounts for only 0.01% of the total area. The total power consumption of the proposed RSFPLL is 8.17 mW. The power breakdown analysis shows that most of the power consumption happens from LC-VCO (42%) and PI/RSPD analog blocks (9.8%), which is understandable. Noticeably, the digital block (DSM, MMDIV, and PSCG) only consumes 0.8 mW (0.7%) of power. This confirms that the proposed fractional-N operation for the RSPLL based on PSCG techniques is area and energy efficient.

Before commenting on the performance of the proposed RSFPLL, we briefly describe the measurement environment. The supply voltage level is 1.2 V from the power supply equipment Agilent E3646A. Reference input sine waves of 100 MHz frequency with low-phase noise are derived from the off-chip Voltage Control Crystal Oscillator (VCXO). Based on the above configuration, the output frequency varies from 2.17 GHz to 2.3 GHz depending on the effective capacitance of the cap bank in VCO.

**Figure 16:**
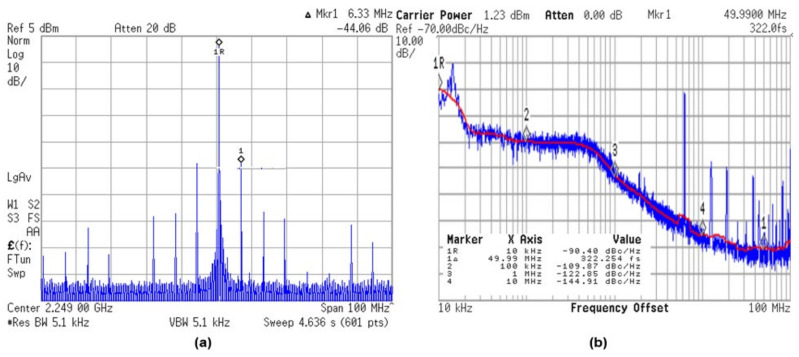
(**a**) Measured output spectrum at 2.249 GHz, (**b**) measured phase noise at 10 kHz to 50 MHz.

Figure 16 shows the measured spurs and phase noise when the PLL performs a fractional-N operation. The measurement results show that the worst fractional spur is about −44.06 dBc, and the integrated jitter value from 10 kHz to 50 MHz is 322 fs, under the following measurement conditions: A VCXO frequency of 100 MHz, a division ratio of 22.5, and a carrier frequency of 2.249 GHz. In-band phase noise value is −109.9 dBc/Hz at 100 kHz and the out-of-band phase noise value is −144.9 dBc/Hz at 10 MHz. The figure-of-merit (FoM) value is calculated as −240.7. Table 1 summarizes detailed performance numbers compared to other fractional-N PLLs.

## 6. Conclusions

A 2.2 GHz reference-sampling fractional-N PLL based on the PSCG technique is proposed. The proposed fractional-N PLL leverages reference-sampling techniques to eliminate reference buffer noise. In RSPLL, a buffered VCO signal samples the input reference signal. Since the VCO-rated sampling consumes enormous power, it is necessary to selectively generate a sampling pulse based on mask window generation and sample only the region of interest. To this end, we proposed a flexible window technique based on the PSCG unit that enables area and energy-efficient fractional-N operation of RSPLL without DTC unit in loops that requires large design efforts. However, implementing a high-resolution fractional-N PLL requires a very precise phase-interpolator. Therefore, the CCS method that makes the PI robust over PVT changes is added to the conventional PI design. Even with all the new features we have achieved, the area overhead of additional digital units is quite small, envisioning an area and energy-efficient RSFPLL compared to the PLL systems in Table 1. The proposed PI-linked RSFPLL is fabricated with a 65 nm CMOS process. The proposed system has an RMS jitter of 322 fs, 8.17 mW of power consumption, and a worst-case spur of −44.06 dBc in the range of 10 kHz to 50 MHz.

## Figures and Tables

**Table 1:** Performance summary and comparison of fractional-N PLL.

	Tao [20] TOCAS-I’19	Chen [26] ISSCC’15	Chang [15] JSSC’14	Liao [27] JSSC’18	Liu [28] TOCAS-I’17	This Work
PLL Type	Sub-Sampling Analog Fractional-N	Sub-Sampling Digital Fractional-N	Sub-Sampling Analog Fractional-N	Sub-Sampling Analog Fractional-N	Sub-Sampling Digital Fractional-N	Ref-Sampling Analog Fractional-N
Technology	130 nm	65 nm	180 nm	130 nm	40 nm	65 nm
Ref. (MHz)	50	49.15	48	50	40	100
Output (GHz)	2.3	2.6~3.9	2.12~2.4	2.39~2.46	1.7~2.7	2.17~2.3
Bandwidth (MHz)	1	-	0.5	1.5	1.5	2
In-band PN (dBc/Hz)	−112 (@200 kHz)	−110.6 (@100 kHz)	−112 (@50 kHz)	−120 (@1 MHz)	−109 (@1 MHz)	−109.9 (@100 kHz)
Worst spur(dBc)	−48.5	−48.5	−48	−52	−37	−44.06
RMS jitter (fs)	358.2 (10 k~10 M)	226 (1 k~100 M)	266 (10 k~30 M)	169 (10 k~10 M)	1700 (10 k~10 M)	322 (10 k~50 M)
Power (mW)	3.2	11.5	17.3	21	1.19	8.17
Area (mm^2^)	0.45	0.23	0.75	0.43	0.22	0.42
* FoM	−244	−241.8	−239.1	−242	−234.6	−240.7

* $\;\mathrm{FoM}=10\;\log_{10}\left(\frac{\sigma_{jitter}^{2}}{1s}\cdot \frac{power}{1mW}\right)$.

## Data Availability

Not applicable.
